# Supplementary material for: The Arabidopsis Cdk1/Cdk2 homolog CDKA;1 controls chromosome axis assembly during plant meiosis
Source: EMBO J. 2019 Sep 26;39(3):e101625. doi: 10.15252/embj.2019101625 (PMC6996576; doi:10.15252/embj.2019101625)
Supplement: Supplementary file 1 — Appendix [file EMBJ-39-e101625-s001.pdf]

Yang et al., Appendix

Table of contents:

Appendix Figures S1-S3

Appendix Table S1

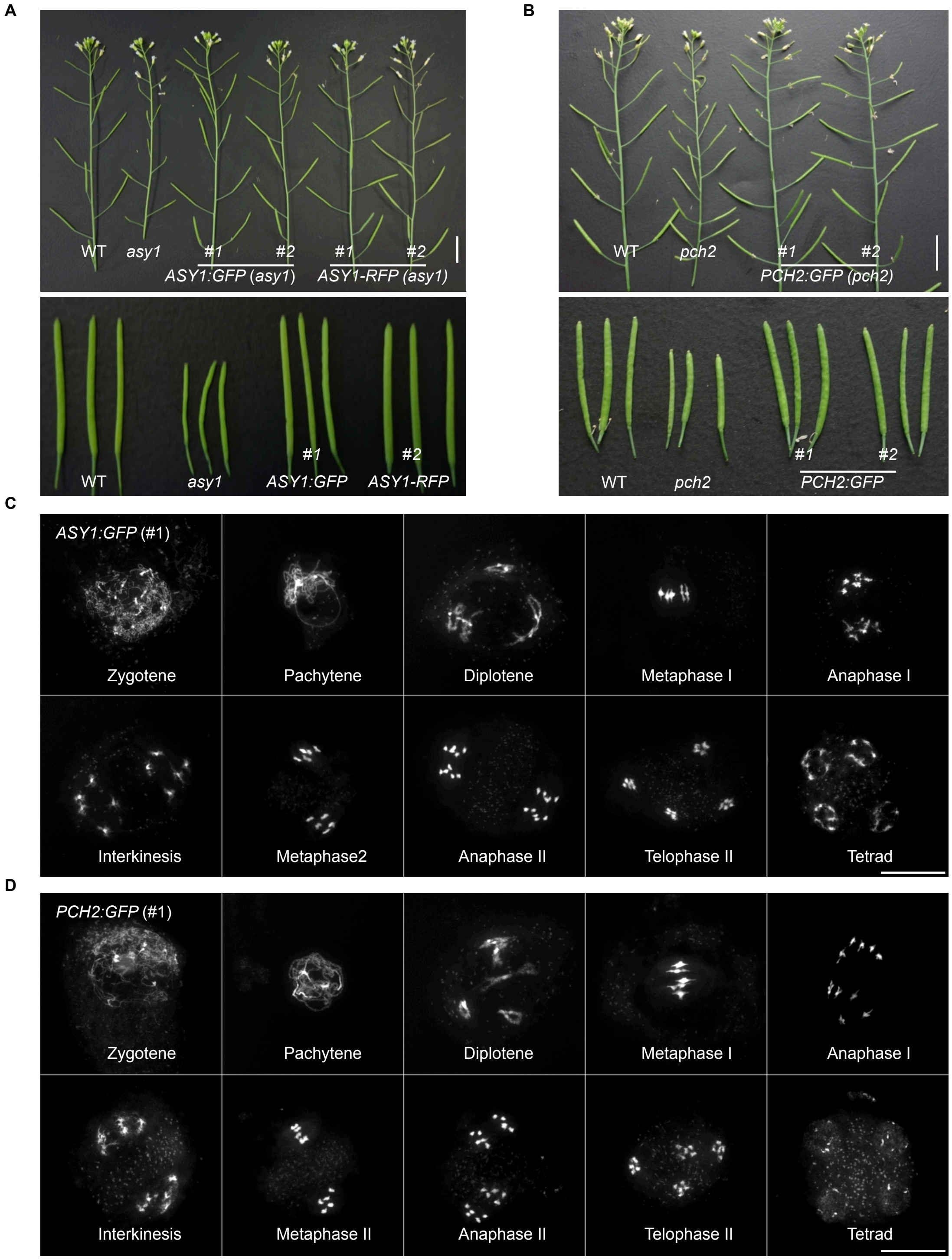

Appendix Figure S1. The ASY1 and PCH2 reporters are fully functional.

(A) The main stems (upper panel) and siliques (lower panel) of the wildtype (WT) , *asy1* and two *ASY1:GFP/RFP* lines are shown.

(B) The main stems (upper panel) and siliques (lower panel) of the wildtype (WT), *pch2* and two *PCH2:GFP* lines.

(C) Chromosome spread analysis of male meiocytes in ASY1:GFP line #*1* shows a wild-type like meiotic program. Bars: 20µm. (D) Chromosome spread analysis of male meiocytes in PCH2:GFP #*1* shows a wild-type like meiotic program. Bars: 20µm.

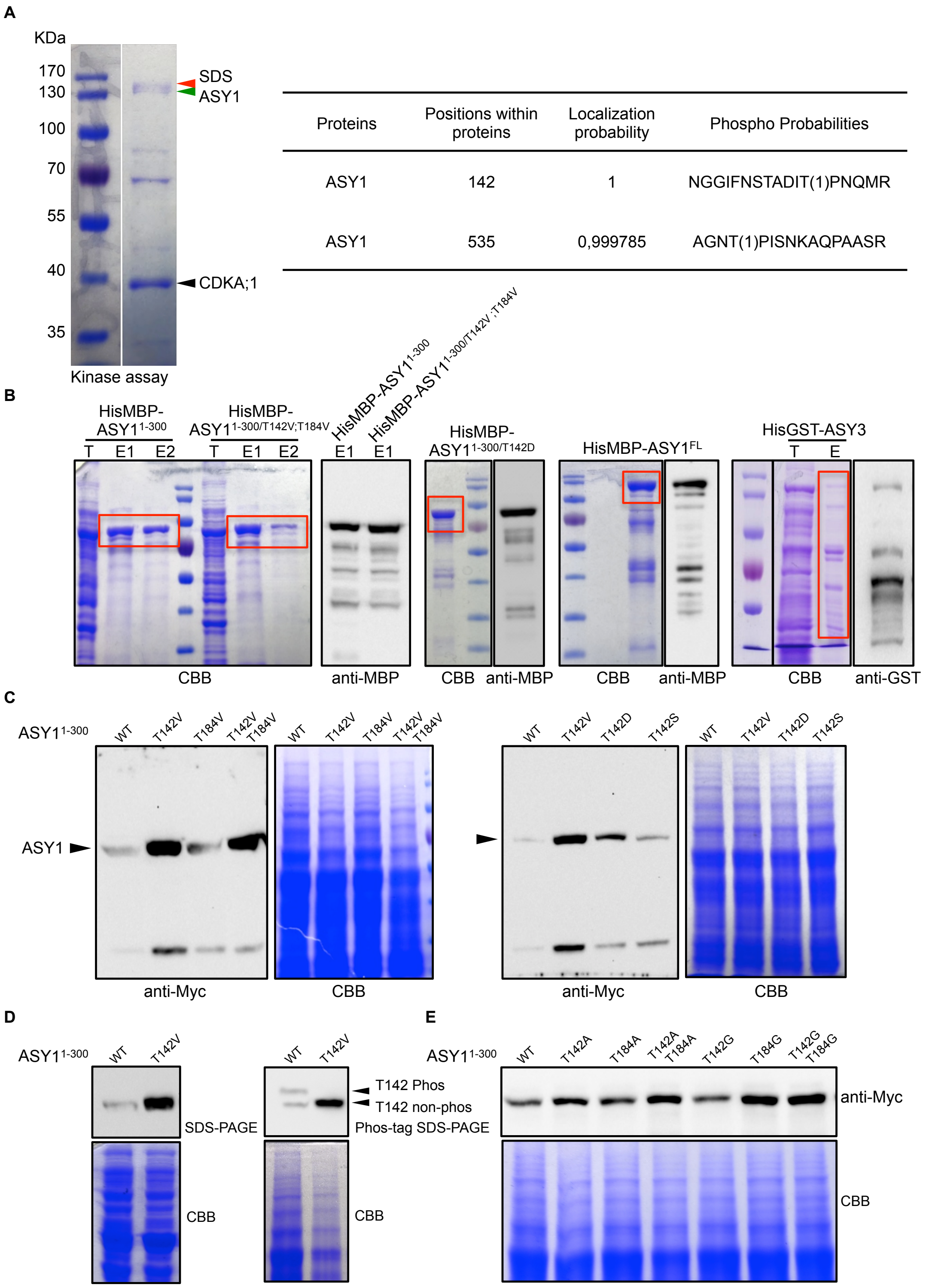

Proteins	Positions within proteins	Localization probability	Phospho Probabilities
ASY1	142	1	NGGIFNSTADIT(1)PNQMR
ASY1	535	0,999785	AGNT(1)PISNKAQPAASR

Appendix Figure S2. Mass spectrometry analysis and coomassie brilliant blue (CBB) stained gels of all purified proteins from *Escherichia coli* used in this research.

(A) CBB staining of the proteins after kinase reaction of ASY1 with CDKA;1-SDS complexes. The red, green and black arrowheads denote the SDS, ASY1, or CDKA;1

proteins, respectively. The table depicts identified ASY1 phosphorylation sites by mass spectrometry, their positions, their localization probabilities and the actual peptides.

(B) CBB staining and western blot of purified HisMBP-ASY1$^{1-300}$, HisMBP-ASY1$^{1-300/T142V;T184V}$, HisMBP-ASY1$^{1-300/T142D}$, HisMBP-ASY1 and His-GST-ASY3 proteins.

(C) Protein abundance analysis of ASY1 variants expressed in yeast. Total protein extracts of yeast cells expressing binding domain-Myc-tagged ASY1$^{1-300}$, ASY1$^{1-300/T142V}$, ASY1$^{1-300/T184V}$, ASY1$^{1-300/T142V;T184V}$, ASY1$^{1-300/T142D}$, and ASY1$^{1-300/T142S}$ were subjected to western blot analysis using an anti-Myc antibody. The protein loading were shown by CBB staining.

(D) The left panel shows the SDS-PAGE analysis of the extracts of yeast cells expressing ASY1$^{1-300}$ and ASY1$^{1-300/T142V}$ and the right panel denotes the phos-tag gel analysis using an anti-Myc antibody. The CBB staining shows the protein loading.

(E) Protein abundance analysis of ASY1 variants expressed in yeast. Total protein extracts of yeast cells expressing binding domain-Myc-tagged ASY1$^{1-300}$, ASY1$^{1-300/T142A}$, ASY1$^{1-300/T184A}$, ASY1$^{1-300/T142A;T184A}$, ASY1$^{1-300/T142G}$, ASY1$^{1-300/T184G}$, and ASY1$^{1-300/T142G;T184G}$ were subjected to western blot analysis using an anti-Myc antibody. The protein loading were shown by CBB staining.

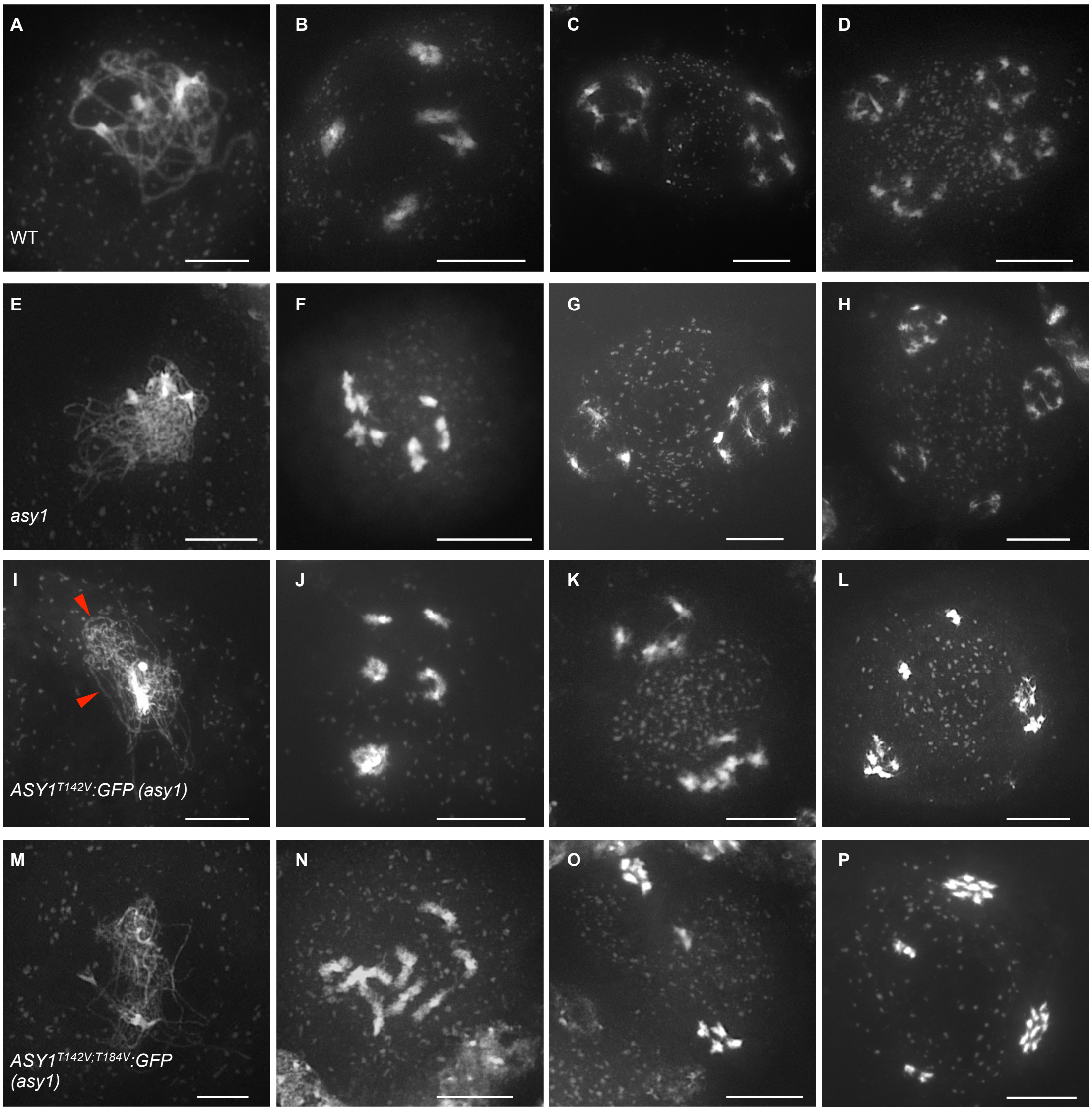

Appendix Figure S3. Chromosome spread analysis of male meiocytes in the wildtype (WT) (A-D), *asy1* mutant (E-H), ASY1^{T142V}:GFP (*asy1*) (I-L), and ASY1$^{T142V;T184V}$:GFP (*asy1*) (M-P) plants.

(A, E, I, M) pachytene or pachytene-like stages; (B, F, J, N) diakinesis or diakinesis-like stages; (C, G, K, O) interkinesis or interkinesis-like stages; (D, H, L, P) tetrad or terad-like stages. Red arrowheads indicate the particially synaptic chromosomes. Bars: 5μm.

Appendix table S1. Primer used in the research

Purpose	Primer name	Sequence
CDKA;1:mVenus reporter	gCDKA;1-F	ACCAAGACACCAAGCGCAA
	gCDKA;1-R	CAGAATGGAAGCGTCTTTGCTT
	pENTR2B-CDKA;1-F	AAGCAAAGACGCTTCCATTCTGCGCGGCCGCACTCGAGATA
	pENTR2B-CDKA;1-F	TTGCGCTTGGTGTCTTGGTGGATCCAGTCGACTGAATTG
	gCDKA;1-SmaISTOP-F	GGGATCTTTCCGTATTTTGGTCATT
	gCDKA;1-SmaISTOP-R	GGGAGGCATGCCTCCAAGATCCTTG
ASY1:GFP and ASY1:RFP reporters	gASY1-F	CAGGGTGGGGTCCAGTTAAG
	gASY1-R	TGTCCACGTAATCCAACGGT
	gASY1-smaISTOP- F	GGGTGAAGACACCACCTCTA
	gASY1-smaISTOP- R	GGGATTAGCTTGAGATTTCTG
	pENTR2B-ASY1-F	TATCAACCGTTGGATTACGTGGACAG CGGCCGCACTCGAGATATC
	pENTR2B-ASY1-R	CTCTTCTTAACTGGACCCCACCCTGG GATCCAGTCGACTGAATTG
ASY1:GFP dephospho- and phospho-mimicry variants	gASY1-T142V-F	GTCCCAAATCAAATGAGGTGC
	gASY1-T142V-R	AATGTCAGCAGTGGAGTTA
	gASY1-T184V-F	GTGGTATGATTACAGCCTTCC
	gASY1-T184V-R	CACATCATCGTAGTACAGAA
	gASY1-T365V-F	GTACCAGAGAGCGAATTCACC
	gASY1-T365V-R	CTGAAATTTTGGGGTAAGTA
	gASY1S382V -F	GTTCCAGGGAAATCTGTTGCT
	gASY1-S382V-R	AATTTGACCATCGGCTTCCT
	gASY1-T535V-F	GTTCCCATTAGCAACAAGGCA
	gASY1-T535V-R	GTTCCCAGCTTTAGAGATAG
	gASY1-T142S-F	TCACCAAATCAAATGAGgtgcagtgttg
	gASY1-T142D-F	GATCCAAATCAAATGAGgtgcagtgttg
ASY1$^{1-570}$:GFP	gASY1-570aa-R	CTGTGAGGCTTGGCTACAGTTGACTGTC
	mGFP-F	CCCGGGGGTGGCatggtgagcaagggcgaggagc
Y2H and/or protein expression	ASY1-attB1-F	GGGGACAAGTTTGTACAAAAAAGCAGGCTTCATGGTGATGGCTC AGAAGCT
	ASY1-attB1-R	GGGGACCACTTTGTACAAGAAAGCTGGGTTTCAATTAGCTTGAG ATTTCTG
	ASY1 1-300 attB2-R	GGGGACCACTTTGTACAAGAAAGCTGGGTTTCACTCAGCCGGAT CCTGTGTGT
	ASY1 T142V CDS-F	GTCCCAAATCAAATGAGGAGTTCAG
	ASY1 T142V CDS-R	AATGTCAGCAGTGGAGTTAAATATT
	ASY1 T184V CDS-F	GTGCCACCAGATTACGAGCCACCTT
	ASY1 T184V CDS-R	CACATCATCGTAGTACAGAAGCTTC
	ASY1-571aa-F	GACAGACGTGGCAGGAAAACCAGCATGG
	attL1-R2	GAAGCCTGCTTTTTTGTACAAAGTTGG
	ASY1-570attB2-R	GGGGACCACTTTGTACAAGAAAGCTGGGTTTCACTGTGAGGCTT GGCTACAGTTG
	ASY1 T142S CDS-F	TCACCAAATCAAATGAGGAGTTCAG
	ASY1 T142D CDS-F	GACCCAAATCAAATGAGGAGTTCAG
PCH2:GFP reporter	gPch2-F	TACATGGAAGCTAAAGTCGTCGTCAG
	gPch2-R	GGGGACAAGTTTGTACAAAAAAGCAGGCTTCACACGGATACTGC CTTCAAGACAA
	gPch2-attB1-F	GGGGACCACTTTGTACAAGAAAGCTGGGTTgatcagatgacttggttgctg ac
	gPch2-interAscI-F	AAGCATGGCGCGCCGCGACTATTCCAGTGCAAATAGCCG
	gPch2-interAscI-R	AAGCATGGCGCGCCTGCTTCAATGGGGTTTTGGTAAGAG

Genotyping of *cdka;1*	CDKA;1-WT-F	AAAAAACTATAACATAATTGGCAAC
	CDKA;1-WT/mut-R	TGTACAAGCGAATAAAGACATTTGA
	CDKA;1-mut-F	GCGTGGACCGCTTGCTGCAACTCTCTCAGG
Genotyping of *asy1*	N546272L	AGGTGGCTCGTAATCTGGTGGCTGC
	N546272U	TCTATGTTTGTTACGCGTTAATCAG
	SALK LBb1.3	ATTTTGCCGATTTCGGAAC
	GenotypeT1 WT allele	CTGGGTTGGGCTGTAACATT
