# Supplementary material for: The Arabidopsis Cdk1/Cdk2 homolog CDKA;1 controls chromosome axis assembly during plant meiosis
Source: EMBO J. 2019 Sep 26;39(3):e101625. doi: 10.15252/embj.2019101625 (PMC6996576; doi:10.15252/embj.2019101625)
Supplement: Supplementary file 2 — Expanded View Figures PDF [file EMBJ-39-e101625-s002.pdf]

Expanded View Figures

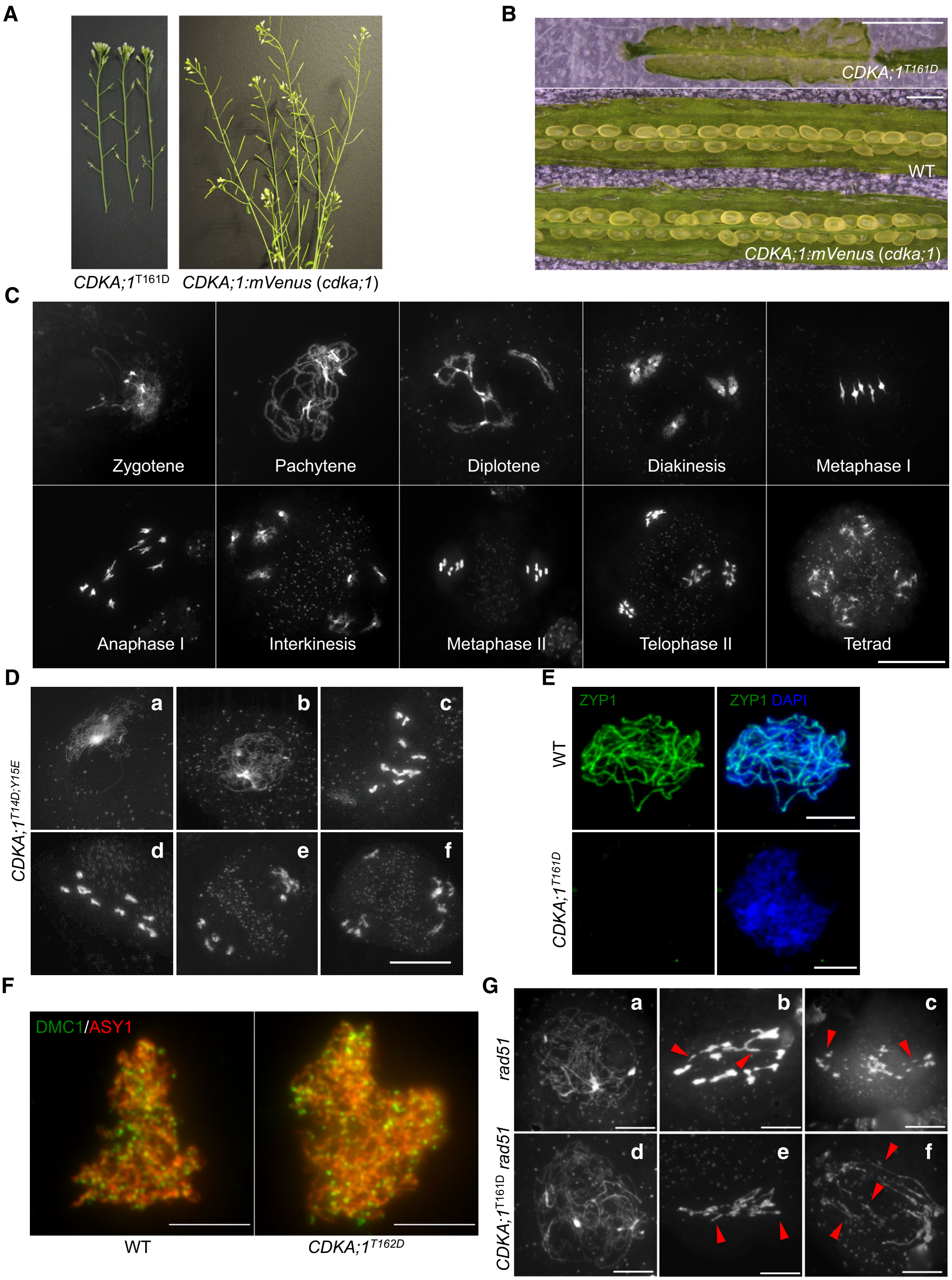

Figure EV1.

© 2019 The Authors

Figure EV1. CDKA;1-mVenus fully complements the *cdka;1* mutant phenotype.

A The stems of a hypomorphic *cdka;1* mutant *CDKA;1*^{T161D} are completely sterile as indicated by short siliques in contrast to homozygous *cdka;1* mutant expressing the *CDKA;1:mVenus* reporter construct that form long siliques and are full fertile.
B The siliques of hypomorphic *CDKA;1*^{T161D} do not harbor viable seeds in contrast to homozygous *cdka;1* mutant expressing CDKA;1:mVenus that develop healthy and plump seeds. Scale bars: 1 mm.
C Chromosome spread analysis of male meiocytes of a homozygous *cdka;1* mutant expressing a functional *CDKA;1:mVenus* reporter reveals a wild type-like meiotic program. Scale bar: 20 μm.
D Chromosome spread analysis of the hypomorphic *cdka;1* mutant *CDKA;1*$^{T14D;Y15E}$. (a) zygotene-like stage; (b) pachytene-like stage; (c, d) diakinesis-like stages; and (e, f) end of meiosis I with two or three pools of chromosomes. Scale bar: 20 μm.
E Immunolocalization of ZYP1 (green) in wild-type (WT) and *CDKA;1*^{T161D} mutants. Chromosomes are stained with DAPI (blue). Scale bars: 5 μm.
F Immunolocalization analysis of DMC1 (green) together with ASY1 (red) in late leptotene of male meiocytes of wild-type (WT) and *CDKA;1*^{T161D} mutants. Scale bars: 5 μm.
G Chromosome spread analysis of *rad51* and *rad51 CDKA;1*^{T161D} mutants. (a, d) pachytene-like stage; (b, c, e, and f) anaphase I-like stage. Red arrowheads indicate the chromosomal fragments. Scale bars: 10 μm.

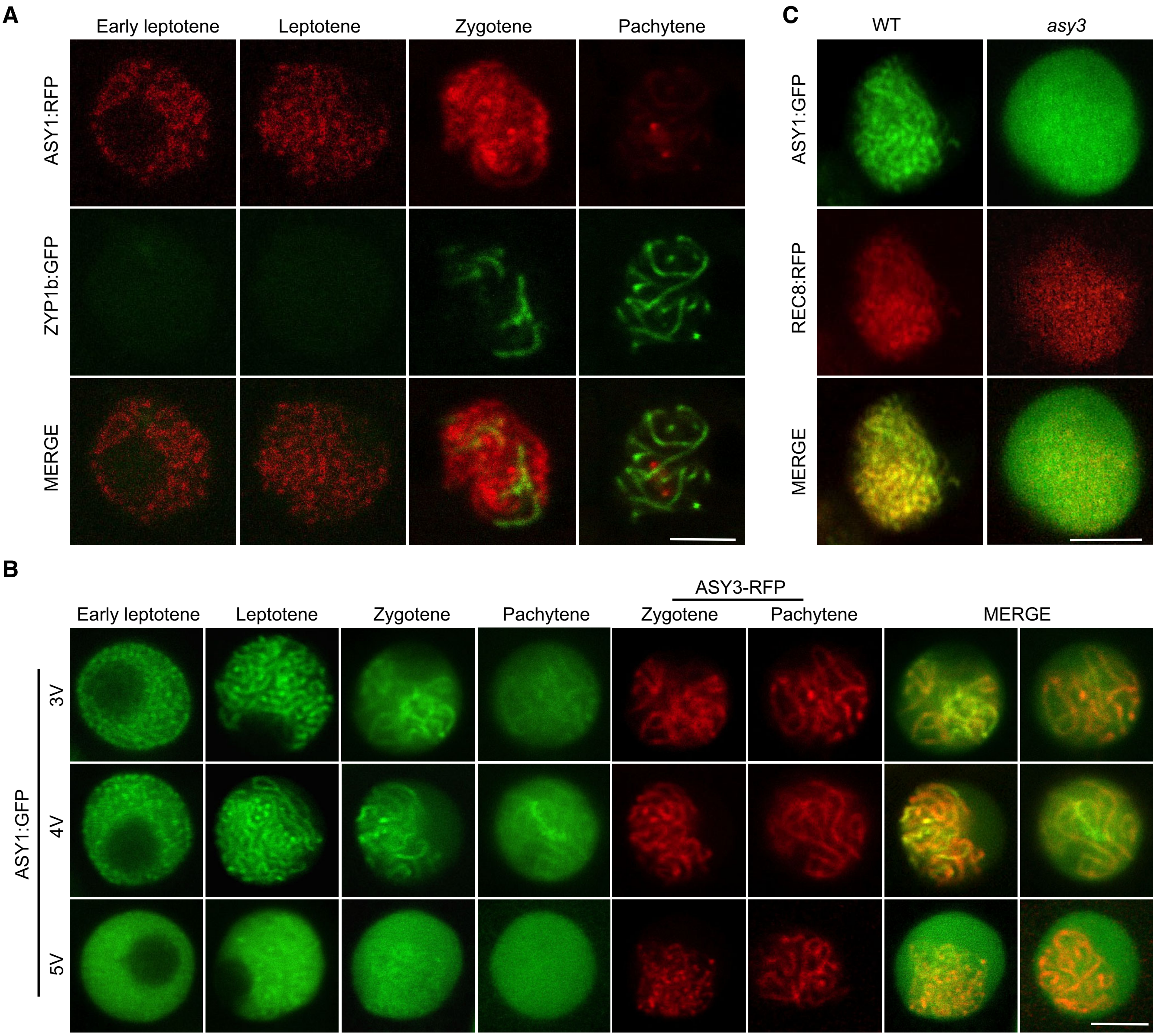

Figure EV2.

Figure EV2. Localization of ASY1 variants in the wild-type and *asy3* mutants.

A Co-localization analysis of ASY1-RFP with ZYP1b-GFP at different meiotic stages in male meiocytes of the wild type. Scale bars: 5 μm.
B Localization of $ASY1^{3V}$:GFP (T365V S382V T535V), $ASY1^{4V}$:GFP (T184V T365V S382V T535V), and $ASY1^{5V}$:GFP (T142V T184V T365V S382V T535V) together with ASY3:RFP (for staging of zygotene and pachytene) at different meiotic stages in male meiocytes of *asy1* mutants. Scale bars: 5 μm.
C Localization of ASY1:GFP in the male meiocytes of the wild-type and *asy3* mutants at leptotene. REC8-RFP was used for staging and to highlight chromosomes. Scale bars: 5 μm.

Figure EV3. Phenotypic characterization of different ASY1:GFP variants.

A Schematic graph showing different ASY1 non-phosphorylatable mutants.
B, C Siliques (B) and seed set (C) of the wild type (WT), *asy1*, $ASY1^{T142V}$, $ASY1^{T184V}$, $ASY1^{2V}$, $ASY1^{3V}$, $ASY1^{4V}$, $ASY1^{5V}$, $ASY1^{T142S}$, and $ASY1^{T142D}$. Red arrowheads indicate aborted seeds.
D Quantification of the seed set shown in (C) from at least five siliques.
E Peterson staining of anthers for the wild type (WT), *asy1*, $ASY1^{T142V}$, $ASY1^{T184V}$, $ASY1^{2V}$, $ASY1^{3V}$, $ASY1^{4V}$, $ASY1^{5V}$, $ASY1^{T142S}$, and $ASY1^{T142D}$. Red indicates viable pollen grains, and blue denotes aborted pollen grains.
F Quantification of the pollen viability assay shown in (E) using at least nine flower buds.

Data information: (D, F) Level of significance ($P < 0.05$) is indicated by different letters as determined by the one-way ANOVA followed by Tukey's test. Error bars represent mean ± SD.

 © 2019 The Authors

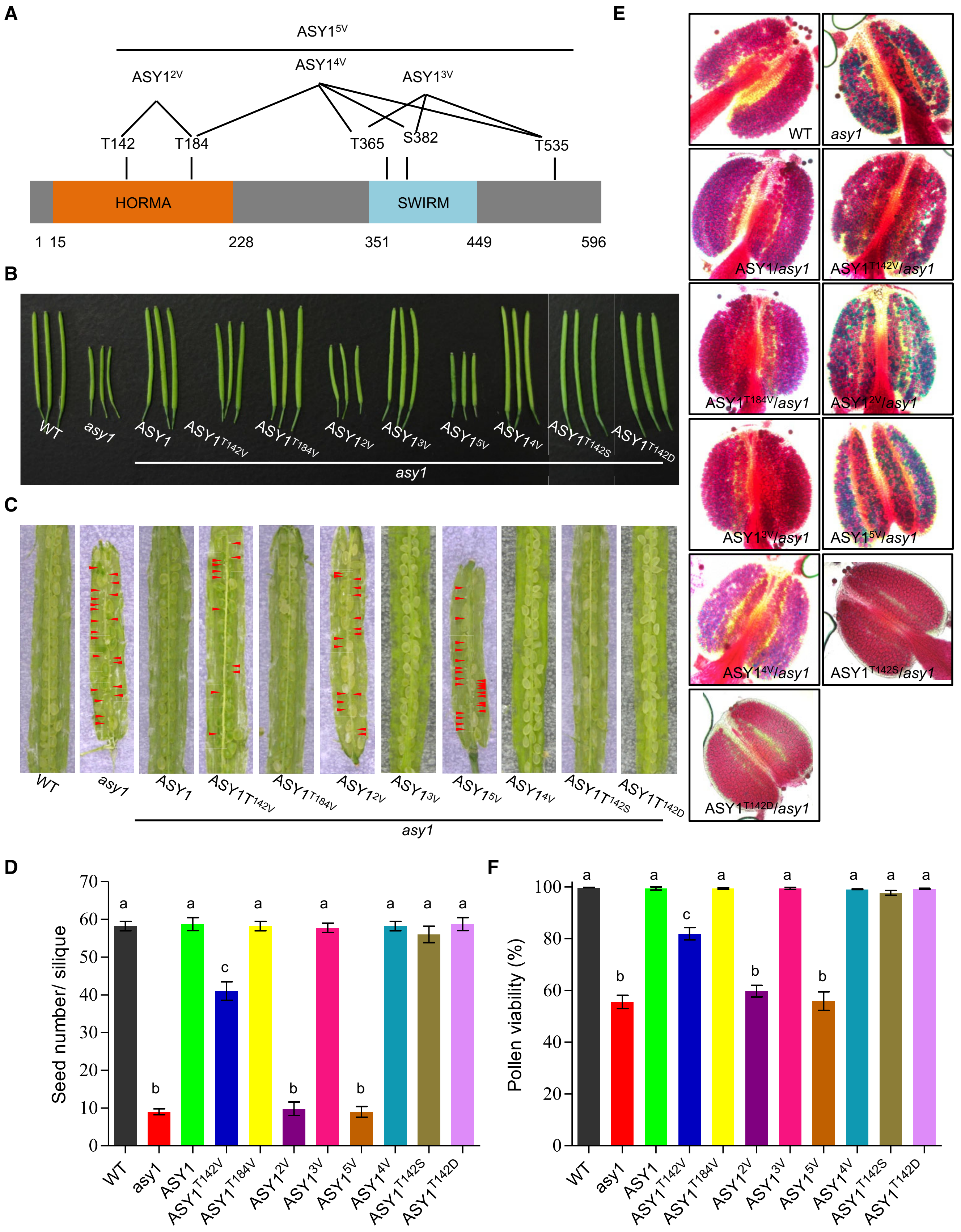
A
ASY1^5V
ASY1^4V
ASY1^2V
ASY1^3V
T142
T184
T365
S382
T535
HORMA
SWIRM
1 15
228
351
449
596
B
WT
asy1
ASY1
ASY1T142V
ASY1T184V
ASY1^2V
ASY1^3V
ASY1^5V
ASY1^4V
ASY1T142S
ASY1T142D
asy1
C
WT
asy1
ASY1
ASY1T142V
ASY1T184V
ASY1^2V
ASY1^3V
ASY1^5V
ASY1^4V
ASY1T142S
ASY1T142D
asy1
D
Seed number/ silique
0
10
20
30
40
50
60
70
a
b
c
E
WT
asy1
ASY1/asy1
ASY1^T142V/asy1
ASY1^T184V/asy1
ASY1^2V/asy1
ASY1^3V/asy1
ASY1^5V/asy1
ASY1^4V/asy1
ASY1^T142S/asy1
ASY1^T142D/asy1
F
Pollen viability (%)
0
20
40
60
80
100

Figure EV3.

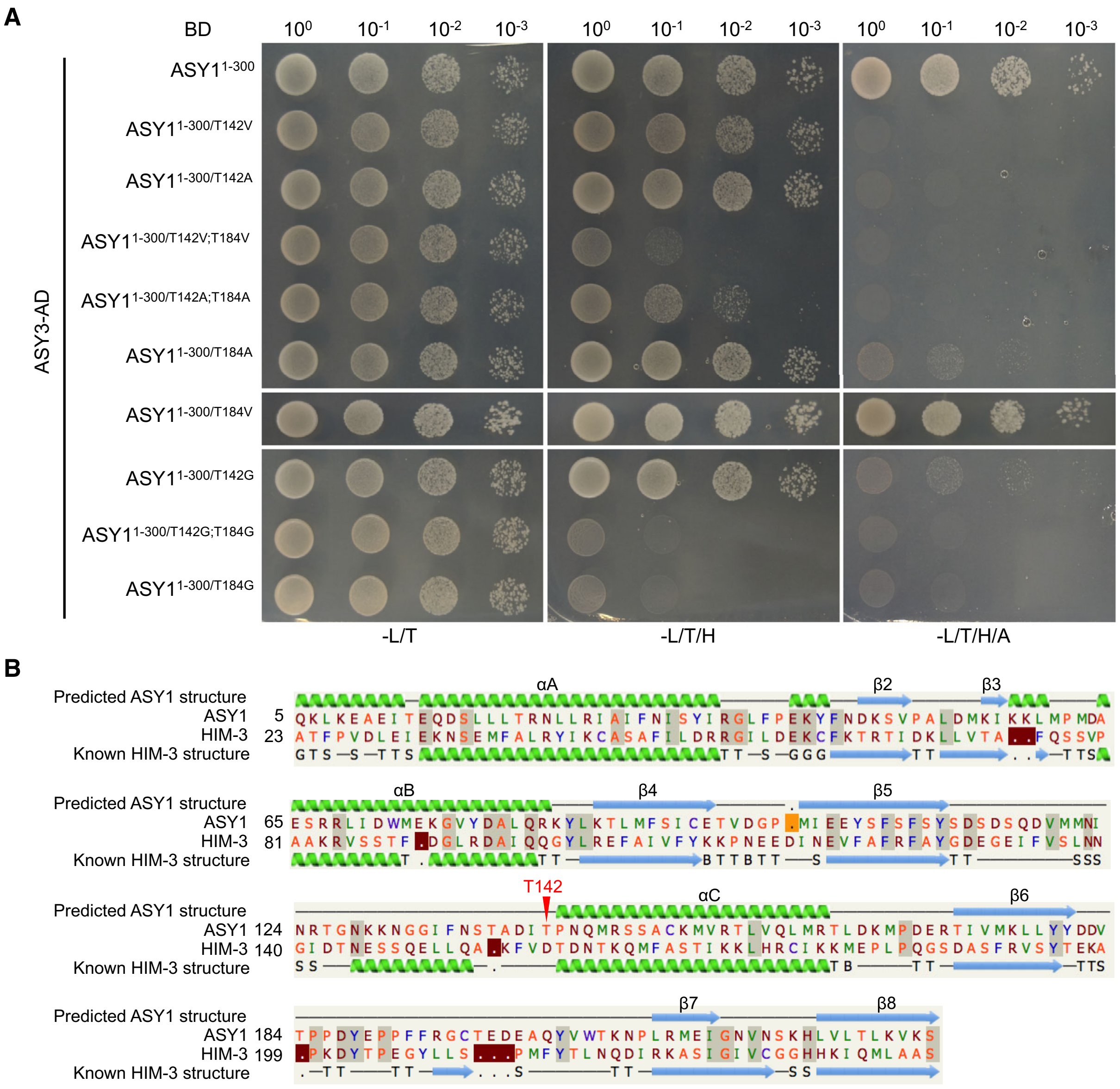

Figure EV4. Non-phosphorylatable substitutions of T142 in ASY1 reduce its interaction strength with ASY3.

A Yeast two-hybrid interaction assays of ASY3 with different ASY1 variants. Yeast cells harboring both the AD and BD plasmids were grown on synthetic medium supplied with glucose in the absence of Leu and Trp (-L/T, left panel), on SD medium in the absence of Leu, Trp, and His (-L/T/H, middle panel), and on SD medium in the absence of Leu, Trp, His, and Ade (-L/T/H/A, right panel). Yeast cells were incubated until OD_{600} = 1 and then diluted 10-, 100-, and 1,000-fold for the assays.

B The predicted structure of ASY1 HORMA domain based on the known structure of *C. elegans* HIM-3 (c4trkA) using Phyre2 protein structure prediction. Red arrowhead indicates the T142 site of ASY1.

 © 2019 The Authors
