# Supplementary figures and images for: The Arabidopsis Cdk1/Cdk2 homolog CDKA;1 controls chromosome axis assembly during plant meiosis
Source: EMBO J. 2019 Sep 26;39(3):e101625. doi: 10.15252/embj.2019101625 (PMC6996576; doi:10.15252/embj.2019101625)

Appendix Figure S2

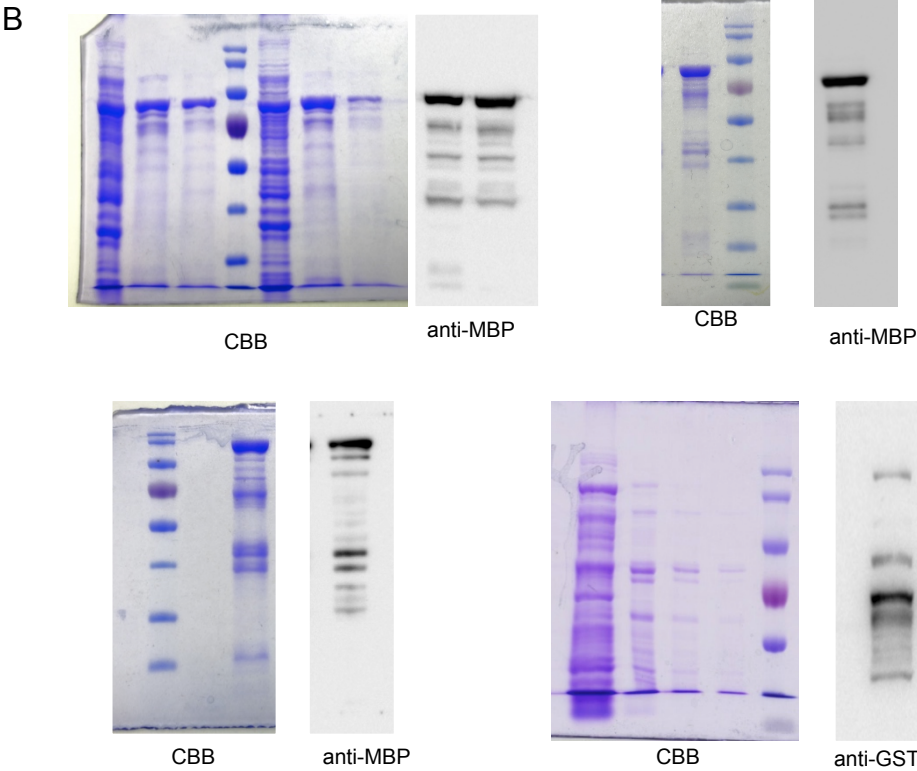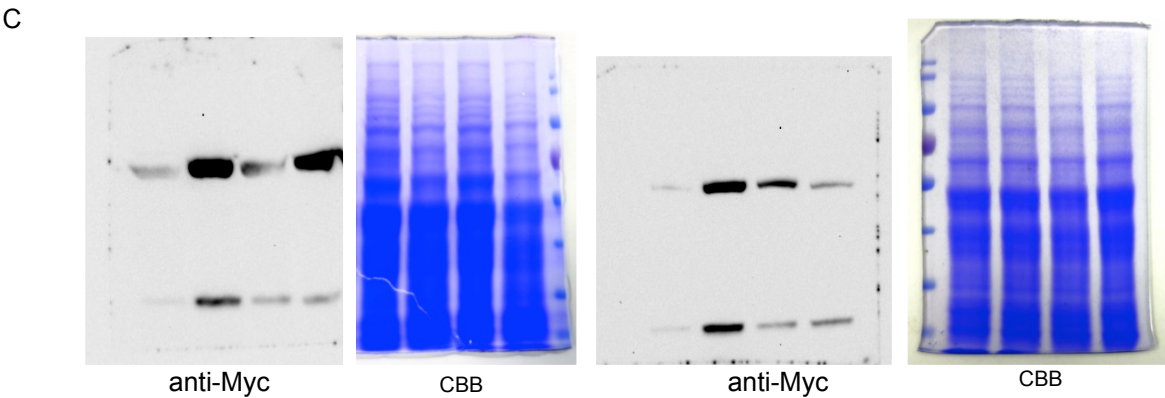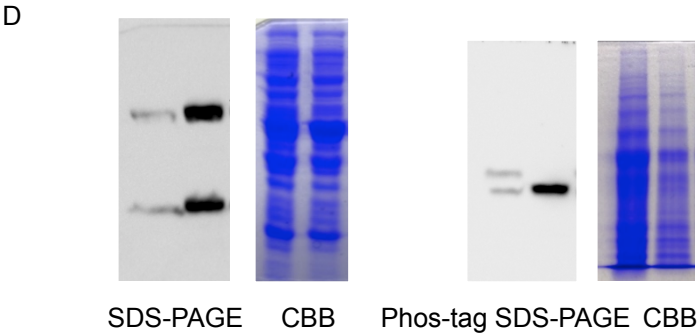

E

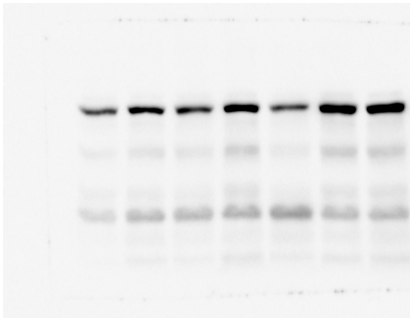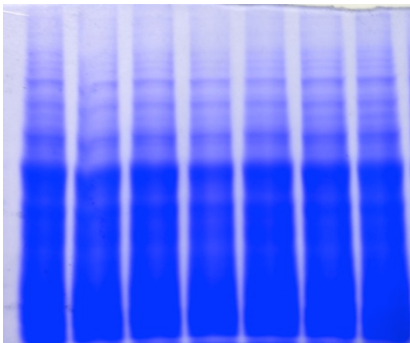

Supplement: Supplementary file 4 — Source Data for Appendix [file EMBJ-39-e101625-s006.pdf]

Figure 2D

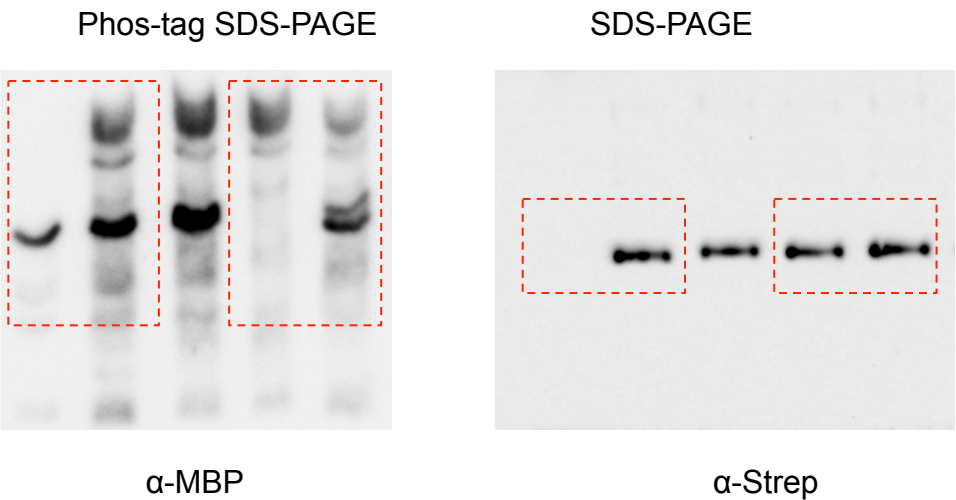

Supplement: Supplementary file 6 — Source Data for Figure 2 [file EMBJ-39-e101625-s004.pdf]

Figure 5B

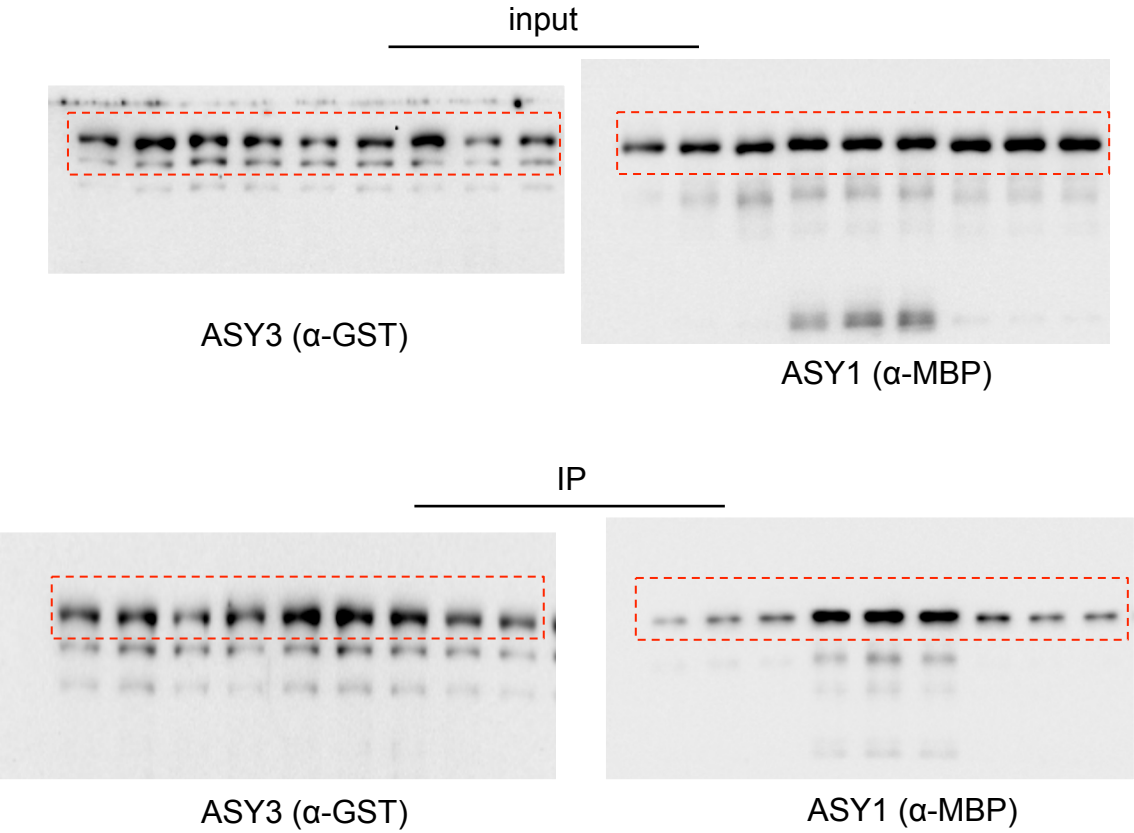

Supplement: Supplementary file 7 — Source Data for Figure 5 [file EMBJ-39-e101625-s005.pdf]
